# A Mare’s Nest

**Published:** 1894-03

**Authors:** 


### A MARE'S NEST.

The *Sanitary Era* for January has a sensation. The editor, in a lengthy and enthusiastic write-up, claims to have discovered that blood will cure anything on earth, except death, and he intimates that he would confidently tackle a mild case of that. Furthermore he says that blood can be preserved alive (i. e., "put up in its own juice," we suppose), notwithstanding the ox or cow be dead long since (and here have we not testimony to—if not the immortality of the soul—at least the immortality of the blood of beasts)? The writer claims that this "preserve" is put up in "a mild but sufficient antiseptic fluid," and under the microscope the corpuscles can be seen alive and kicking; further,—he claims that preserved blood—bottled life he calls it—can now be had at any drug store, and its powers are so wonderful and diversified that it is only necessary to get some of it into the system of the patient, by any manner or means whatever, and no matter what be the trouble or how far gone the patient, when lo! he takes up his bed and walks. The enthusiastic discoverer proposes a "Blood Therapy" or "Hæmatherapy," and says in effect that blood is the only really essential article in his new materia medica ! The bug under the chip only crops out when the writer admits that there is a preparation of *ox blood* in the market (for an account of which see the *Era's* advertising pages) which contains all the essentials of live blood,—bottled life;—truly, a wonderful discovery !

So enthused is the *Era* editor over his discovery, and so unselfish, that he writes us that we should take time by the forelock and go in early and avoid the rush; that to get reputation out of the find we should catch on early. Well,—thanks. We remember a good (and unselfish) little boy, to whom mother sought to give some dover's powders disguised with some preserve syrup, and the good doctor, to help matters, said "now, mamma, don't give Johnny all that nice preserve; save some for me." Johnny, in the generosity of his pure young heart, exclaimed "Mamma, doctor's a good man; give him *all* of it!"—We give the editor credit for honesty and sincerity but we suspect he is over-credulous aud has been talked to by some shrewd sample agent.

